# Primary health care and achieving universal health coverage: An emphasis on the crucial role of E-Health

**DOI:** 10.34172/hpp.42933

**Published:** 2024-03-14

**Authors:** Haidar Nadrian

**Affiliations:** Social Determinants of Health Research Center, Tabriz University of Medical Sciences, Tabriz, Iran

 Primary healthcare serves as the foundation of a healthcare system, providing essential and accessible services to individuals and families in their communities. Universal health coverage aims to ensure that all individuals and communities have access to quality healthcare services without facing financial hardship.^1^ Primary healthcare plays a key role in achieving this goal by serving as the first point of contact for individuals seeking healthcare services. By providing a wide range of services, such as preventive care, treatment of common illnesses, and management of chronic conditions, primary healthcare helps to address the healthcare needs of individuals at the community level.

 Family health is closely linked to primary healthcare, as it focuses on the health and well-being of families as a whole. Primary healthcare providers play a critical role in promoting family health by providing comprehensive care to individuals of all ages, from infants to the older adults. By addressing the healthcare needs of families holistically, primary healthcare helps to improve health outcomes and quality of life for individuals and communities. In order to achieve universal health coverage and promote family health, it is essential to strengthen primary healthcare services and ensure their accessibility and affordability for all individuals.^2^ This includes investing in primary healthcare infrastructure, training healthcare providers, and implementing policies that prioritize E-health solutions as a key component of the healthcare system.^3^

 In today’s digital age, technology has revolutionized the way healthcare services are delivered and accessed, making it essential to integrate E-health into primary healthcare systems. E-health encompasses a wide range of electronic tools and technologies that support the delivery of healthcare services, such as electronic health records, telemedicine, and mobile health applications.^4^ These tools have the potential to improve access to healthcare services, enhance communication between healthcare providers and patients, and streamline the delivery of care.^5^

 Electronic referral systems also play a pivotal role in facilitating the coordination of care between primary healthcare providers and specialists, ensuring that patients receive timely and appropriate treatment. By enabling secure and efficient communication between healthcare providers, electronic referral systems help to reduce wait times, improve patient outcomes, and enhance the overall quality of care.^6^

 Integrating e-health solutions and electronic referral systems into primary healthcare can help to overcome barriers to access, improve the efficiency of healthcare delivery, and enhance the coordination of care for individuals and families.^4-6^ Leveraging technology to support primary healthcare services strengthens the foundation of healthcare systems and the works towards achieving universal health coverage and promoting family health. Therefore, investing in the development and implementation of e-health solutions and electronic referral systems seems to be essential to maximize their potential in enhancing primary healthcare and improving health outcomes for individuals and communities. Taking into account technology and innovation in healthcare delivery seems to create a more connected, efficient, and patient-centered healthcare system that meets the diverse needs of populations. As instances, telemedicine and mobile health applications can expand access to healthcare services, particularly in remote and underserved areas of developing countries.^7^

 To fully realize the potential of e-health and electronic referral systems in the health system of developing countries, policymakers must prioritize investment in digital infrastructure, training healthcare providers in the use of technology, and implementing policies that support the integration of e-health solutions into primary healthcare services. Collaboration between government agencies, healthcare providers, and technology companies will be essential to ensure the successful implementation and adoption of e-health solutions.^8^ Eventually, with a focus on the significant role of E-health in achieving the goals of primary healthcare, family health and universal health coverage, we can work towards building a more equitable and inclusive healthcare system that meets the needs of all individuals and communities.

## Competing Interests

 Haidar Nadrian is Associate Editor in Health Promotion Perspectives.

## Ethical Approval

 Not applicable.
